# The analysis of *C9orf72* repeat expansions in a large series of clinically and pathologically diagnosed cases with atypical parkinsonism^☆^

**DOI:** 10.1016/j.neurobiolaging.2014.08.024

**Published:** 2015-02

**Authors:** Lucia V. Schottlaender, James M. Polke, Helen Ling, Nicola D. MacDoanld, Arianna Tucci, Tina Nanji, Alan Pittman, Rohan de Silva, Janice L. Holton, Tamas Revesz, Mary G. Sweeney, Andy B. Singleton, Andrew J. Lees, Kailash P. Bhatia, Henry Houlden

**Affiliations:** a Department of Molecular Neuroscience, UCL Institute of Neurology, The National Hospital for Neurology and Neurosurgery, London, UK; b Neurogenetics Laboratory, UCL Institute of Neurology, The National Hospital for Neurology and Neurosurgery, London, UK; c Reta Lila Weston Institute for Neurological Studies, The Queen Square Brain Bank, UCL Institute of Neurology, The National Hospital for Neurology and Neurosurgery, London, UK; d The MRC Centre for Neuromuscular Diseases, UCL Institute of Neurology, The National Hospital for Neurology and Neurosurgery, London, UK; e Laboratory of Neurogenetics, NIA, NIH, Bethesda, MD, USA; f The Sobell Department of Movement Disorders, UCL Institute of Neurology, The National Hospital for Neurology and Neurosurgery, London, UK

**Keywords:** *C9orf72*, Parkinsonism, Multiple system atrophy (MSA), Progressive supranuclear palsy (PSP), Corticobasal degeneration (CBD) and corticobasal syndrome (CBS)

## Abstract

A GGGGCC repeat expansion in the *C9orf72* gene was recently identified as a major cause of familial and sporadic amyotrophic lateral sclerosis and frontotemporal dementia. There is suggestion that these expansions may be a rare cause of parkinsonian disorders such as progressive supranuclear palsy (PSP), multiple system atrophy (MSA), and corticobasal degeneration (CBD). Screening the *C9orf72* gene in 37 patients with features of corticobasal syndrome (CBS) detected an expansion in 3 patients, confirmed by Southern blotting. In a series of 22 patients with clinically diagnosed PSP, we found 1 patient with an intermediate repeat length. We also screened for the *C9orf72* expansion in a large series of neuropathologically confirmed samples with MSA (n = 96), PSP (n = 177), and CBD (n = 18). Patients were found with no more than 22 GGGGCC repeats. Although these results still need to be confirmed in a larger cohort of CBS and/or CBD patients, these data suggest that in the presence of a family history and/or motor neuron disease features, patients with CBS or clinical PSP should be screened for the *C9orf72* repeat expansion. In addition, we confirm that the *C9orf72* expansions are not associated with pathologically confirmed MSA, PSP, or CBD in a large series of cases.

## 1 Introduction

The identification of a GGGGCC repeat expansion in the *C9orf72* gene (OMIM *614260) has been an important breakthrough in the diagnosis and understanding of neurodegenerative disorders. Expansions in this gene have primarily been identified in familial and sporadic amyotrophic lateral sclerosis (ALS; OMIM #105400) and frontotemporal dementia (FTD; OMIM #600274) (DeJesus-Hernandez et al., 2011; Renton et al., 2011). A greater frequency of expansions has been observed in the Finnish and Northern European populations (Mok et al., 2012). Neuropathologically, TAR DNA-binding protein-43 and p62 inclusions are present in samples carrying *C9orf72* expansions but our knowledge of the neuropathology associated with this genetic abnormality is expanding (Bieniek et al., 2013; Neumann, 2013).

Progressive supranuclear palsy (PSP; OMIM #601104), multiple system atrophy (MSA; OMIM #146500), and corticobasal degeneration (CBD) are collectively referred as atypical parkinsonian disorders, and they each have specific and validated diagnostic criteria (Armstrong et al., 2013; Gilman et al., 2008; Litvan et al., 1996). Patients with MSA present with a combination of cerebellar dysfunction, parkinsonism, and autonomic dysfunction and are pathologically characterized by alpha-synuclein immunoreactive glial cytoplasmic inclusions and neuronal loss in the olivopontocerebellar and striatonigral systems (Ahmed et al., 2012). Postural instability and supranuclear gaze palsy are the salient features in PSP, which exhibits predominantly 4-repeat tau in a characteristic distribution including tufted astrocytes and coiled bodies (Williams and Lees, 2009). CBD is pathologically characterized by cortical and striatal 4-repeat tau deposition with astrocytic plaques being the pathologic hallmark (Ling et al., 2010). Corticobasal syndrome (CBS) consists of a constellation of extrapyramidal and frontoparietal cortical features. CBS is the classic clinical presentation of CBD, but many CBS cases turn out to have alternate neuropathology.

The genetic understanding of the atypical parkinsonian disorders, MSA, PSP, and CBD has lagged behind the commoner neurodegenerative conditions of ALS, FTD, and Parkinson's disease (PD). MSA has been linked to variants in the *SNCA* gene (Al-Chalabi et al., 2009; Kiely et al., 2013; Ross et al., 2010; Scholz et al., 2009). More recently, variants in *COQ2* has been proposed as a cause of familial MSA and a risk factor for sporadic clinically diagnosed MSA in the Japanese population (Multiple-System Atrophy Research Collaboration, 2013), which has failed to be replicated so far (Jeon et al., 2014; Schottlaender et al., 2014; Sharma et al., 2014). Further work could not identify MSA risk genes (Segarane et al., 2009), and a genome-wide association study in MSA is ongoing (Sailer A, personal communication). A genome-wide association study in PSP has confirmed an association with variants in *MAPT* and has shown a significant signal with variants in *EIF2AK3*, *STX6*, and *MOBP* (Höglinger et al., 2011), but further research is needed to understand these findings. The H1/H1 *MAPT* haplotype is associated with PSP and CBD and likewise *MAPT* variants such as p.N410H (Kouri et al., 2014) and p.A152T have been linked to pathologically confirmed CBD and PSP (Houlden et al., 2001; Kara et al., 2012).

Overlap of the clinical features of PSP and CBS are not uncommon, although CBD can have a clinical phenotype of classic PSP, which is known as Richardson syndrome, and rarely PSP may present with CBS (Ling et al., 2010, 2014). In addition, atypical parkinsonian disorders and PD often cluster in pedigrees; however, this is not often inherited in a dominant fashion.

Taking a good family history is essential in the diagnosis of neurodegenerative diseases. Patients with ALS that carry *C9orf72* expansions commonly have a history of FTD and/or a family history of other neurodegenerative or neuropsychiatric disorder (Byrne et al., 2012, 2013; Fallis and Hardiman, 2009). Numerous ALS and FTD series have now been screened for *C9orf72* expansions (Rademakers, 2012). The expansions have also been rarely identified in PD, CBS, PSP, MSA-cerebellar type, and dementia with Lewy bodies (Dejesus-Hernandez et al., 2013; Lesage et al., 2013; Lindquist et al., 2013; Snowden et al., 2012; Ticozzi et al., 2014). The expansion has also been detected in some families with Alzheimer's disease (AD) but few had pathologic confirmation (Beck et al., 2013; Harms et al., 2013; Majounie et al., 2012; Xi et al., 2012). *C9orf72* expansions have been identified in nondemented healthy elderly individuals, suggesting incomplete penetrance (Beck et al., 2013; Cooper-Knock et al., 2013; Galimberti et al., 2014).

Based on the large heterogeneity of patients with a *C9orf72* repeat expansion we aim to assess the frequency of this genetic variation in a large series of patients with atypical parkinsonism.

## 2 Methods

### 2.1 Informed consent and standard protocol approvals

Informed consent was obtained for genetic analysis from all patients. Brain tissue from neuropathologically confirmed samples was obtained from the Queen Square, Harvard, Maryland and the Netherlands Brain Banks (UCLH ethics approval (UG2UPD|04/Q0505/2). Tissue is stored in the Queen Square Brain Bank (QSBB) under a license from the Human Tissue Authority and has been donated for research according to protocols approved by the NRES committee London-Central.

### 2.2 Sample and DNA extraction

Screening for the *C9orf72* expansion was performed in both clinically diagnosed atypical parkinsonism patients as well as pathologically confirmed samples. The clinically diagnosed cohort included CBS (n = 37) and PSP (n = 22) samples, and the pathologically confirmed series comprised MSA (n = 96), PSP (n = 177), and CBD (n = 18) samples.

Genomic DNA was extracted from peripheral blood using Flexigene extraction kit and Autopure LS extraction system (Qiagen, Venlo, the Netherlands) and from brain tissue using the DNeasy Tissue Kit (Qiagen, Hilden, Germany) according to the manufacturer's instructions.

### 2.3 Repeat-primed polymerase chain reaction

To provide a qualitative assessment of the presence of an expanded GGGGCC hexanucleotide repeat in *C9orf72*, we performed a repeat-primed polymerase chain reaction (PCR). The repeat was amplified with a PCR reaction performed in the presence of 1 M betaine, with Extensor long PCR master mix (Thermo Scientific), using a previously described cycling program (Gijselinck et al., 2012). Primer sequences using one fluorescently labeled primer were used as previously published (DeJesus-Hernandez et al., 2011). A second sizing PCR was also used to size alleles of <30 repeats. This PCR used previously published primers (DeJesus-Hernandez et al., 2011) in a reaction with Extensor long PCR master mix (Thermo Scientific) supplemented with betaine, dimethyl sulfoxide, magnesium chloride, and 7-deaza-2-deoxy GTP. All PCR products were analyzed by fragment length analysis on an automated ABI3730 DNA-analyzer, and allele identification and scoring was accomplished using GeneMapper v3.7 software (ABI).

### 2.4 Southern blotting

Confirmation of expansions and intermediate repeats was performed by Southern blotting with a 1 kb single copy probe as previously described (Fratta et al., 2013) but using BsU36I or BamHI/EcoRI restriction enzyme digests that generate a 6.2 kb or 2.4 kb band for unexpanded alleles, respectively, rather than the EcoRI digest used previously that generates an 8 kb band.

## 3 Results

**Fig. 1:**
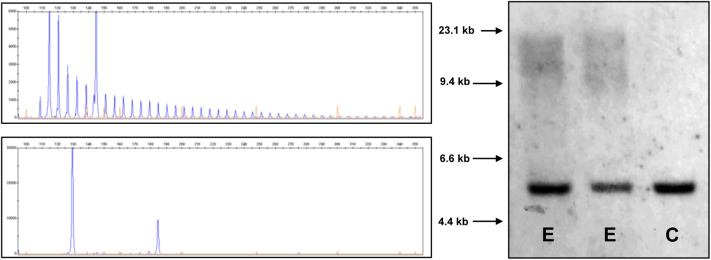
Example of fragment analysis in *C9orf72*. Upper left figure showing heterozygous expansion by RP-PCR, lower left showing unexpanded allele by sizing PCR. Figure on the right is a Southern blot (with BsU36I restriction digest) showing 2 of the expanded CBS cases. Abbreviations: C, control no expansion; CBS, corticobasal syndrome; E, expanded; PCR, polymerase chain reaction; RP-PCR, repeat-primed polymerase chain reaction.

**Fig. 2:**
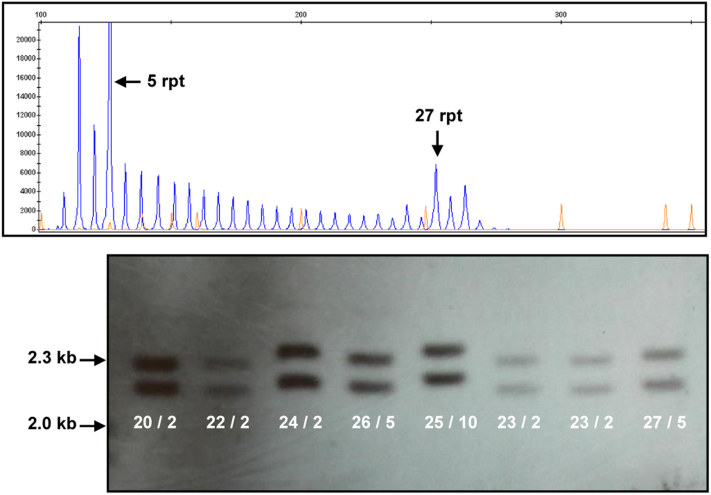
Top figure: RP-PCR showing the case with heterozygous 27 repeats on 1 allele, 5 repeats on the other. Bottom figure: Southern blot confirmation (with BamHI/EcoRI double-digest) of different repeat sizes from 20 to 27 showing the Southern blot appearance of different fragments. Numbers = number of *C9orf72* repeats. Number of repeats was also confirmed by fluorescent PCR of the *C9orf72* repeat and fragment analysis. Abbreviations: PCR, polymerase chain reaction; RP-PCR, repeat-primed polymerase chain reaction.

Among clinically diagnosed patients, a pathologic expansion in *C9orf72* was detected in 3 CBS patients, representing a significant association when compared with published British controls (*p* < 0.001) (Beck et al., 2013) (Fig. 1, Table 1). A single patient with clinically diagnosed PSP was found to carry an intermediate allele of 27 repeat length in *C9orf72* (Fig. 2). All these expansions were confirmed by Southern blot (Figs. 1 and 2).

All pathologically confirmed MSA, PSP, and CBD samples were found not to have pathologic expansions. No patient had more than 22 GGGGCC *C9orf72* repeats (range, 2–22 repeats).

Clinically, the 3 patients that carry a large *C9orf72* expansion presented with a CBS phenotype classified as probable CBD according to consensus criteria. One of them had additional motor-neuron features and the other 2 had dysphagia and dysarthria as salient features. Two expansion carriers had a family history of dementia. The patient with a 27-repeat allele had an onset later in life, presented and progressed as typical PSP, and had a family history of dementia as well as Parkinson's disease.

**Fig. 3:**
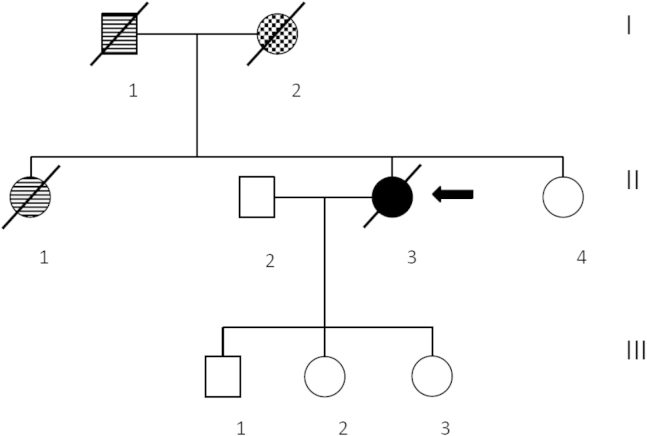
Family tree of the patient (II-3, arrow) with a 27-repeat allele. The patient's father (I-1) and sister (II-1) were diagnosed with dementia, and the patient's mother (I-2) was diagnosed with Parkinson's disease. The clinical features are discussed further in Table 2 and in the text.

The full clinical characteristics of the patients with expansions are described in Table 2. The family tree of the patient with an intermediate repeat is presented in Fig. 3.

## 4 Discussion

In this study, we identified a *C9orf72* expansion in 3 patients with a clinical history consistent with CBS and 1 intermediate expansion in a patient with a clinical history of PSP. The identification of CBS patients with *C9orf72* repeat expansions is an important point to portray to clinicians and diagnostic laboratories. A positive family history was present in 3 of 4 of these patients. This suggests the diagnostic workup of CBS patients with a possible and/or probable family history, in the presence of motor-neuron features, should include screening for *C9orf72* expansions (Ticozzi et al., 2014). The counseling of other family members will be an important issue to discuss in depth given the reduced penetrance and the very rare identification of the expanded repeat in elderly controls (Beck et al., 2013; Galimberti et al., 2014). Furthermore, *C9orf72* repeat expansions may have relevance in patients with CBS and other neurodegenerative disorders, which can have heterogeneous underlying neuropathology (Kouri et al., 2011; Ling et al., 2010). The expansion should also be screened in patients with familial AD as FTD may have an amnestic AD-like presentation.

In addition, we found the absence of *C9orf72* repeat expansions in the largest series of pathologically confirmed MSA, PSP, and CBD samples screened to date. This adds to our knowledge of this genetic abnormality and helps to define the spectrum of *C9orf72* expansion-associated neuropathology. Other unknown and likely poorly penetrant genetic variations consisting of SNPs or repeat expansions in other genes may still be associated with these disorders, although any new genetic finding in MSA would probably differ from PSP and CBD in view of their pathologic differences.

The pathologic cutoff for *C9orf72* repeat expansions remains debatable. Repeat sizes between 20 and 30 are commonly referred to as intermediate alleles and are of uncertain significance; although above 30 repeats are frequently considered pathologic. FTD patients with 20–22 repeats have been reported in the past without any significant clinical difference in the FTD phenotype compared with those with large expansion (Gómez-Tortosa et al., 2013). The *C9orf72* intermediate repeat copies were found to be a significant risk factor for PD in a Spanish study (Nuytemans et al., 2013) but later could not be confirmed in a pathologically confirmed PD cohort (Nuytemans et al., 2014). There is also debate in the field that expansions in *C9orf72* are actually disease modifiers and not disease causative. This belief is reinforced by the fact that double mutants have been identified (Ferrari et al., 2012; King et al., 2013; Mignarri et al., 2014; van Blitterswijk et al., 2013). It is also important to this study that *MAPT* has been found mutated in PSP and *GRN* in CBS—both of which are FTD-associated genes (Benussi et al., 2009; Dopper et al., 2011; Höglinger et al., 2011). Defects in these genes in our cases had been previously excluded.

In this study, the significance of the individual with an intermediate allele of 27 repeats in *C9orf72* is unknown, although the late onset PSP phenotype and the family history may be suggestive of pathogenicity. Unfortunately, we are unable to investigate segregation in this family because of lack of DNA in the deceased patients.

The issue of penetrance, intermediate alleles, and the true pathologic cutoff will be of significant importance in the future from a diagnostic point of view. Predictive counseling of unaffected individuals in families will rely on a future understanding of the mechanistic basis of the expansion and the differences in risk conferred by repeat size variation.

## Disclosure statement

The authors have no conflicts of interest or financial disclosures.

## Figures and Tables

**Table 1:** Results of *C9orf72* repeat expansion screening in atypical parkinsonism

Diagnosis	Number of samples	Expanded
MSA (pathologically confirmed)	96	0
CBD (pathologically confirmed)	18	0
PSP (pathologically confirmed)	177	0
CBS (clinical)	37	3 (*p* < 0.001^a^)
PSP (clinical)	22	1 (27 repeats)
British controls (clinical)	7579	11

Key: CBD, corticobasal degeneration; CBS, corticobasal syndrome; MSA, multiple system atrophy; PSP, progressive supranuclear palsy.

a Fisher exact test comparing our CBS cohort with previously published British controls (Beck et al., 2013).

**Table 2:** Clinical characteristics of the CBS patients with expansions in the *C9orf72* gene, and the clinically diagnosed PSP patient with an allele of 27 repeats

Age of onset, gender	Repeat	Initial presentation	Previous psychiatric features	Other clinical features	Working diagnosis	CBD variant (consensus criteria)	Family history	Imaging
51, F	Expanded	Falls and personality change	Depression	Akinetic-rigid syndrome with cognitive decline and asymmetrical upper-motor neuron signs. Progressed with minimal L-dopa response and atrophy on MRI brain scan.	CBS	Probable-CBS	Father with dementia in his early 50s	MRI: generalized volume loss. DATSCAN: bilateral severe deficit of presynaptic dopamine transporter in the basal ganglia.
44, F	Expanded	Parkinsonism after head trauma	No	Atypical PD with cognitive decline after 9 y, asymmetrical limb manifestations, falls, hypometric saccades and frontal liberation signs, dysphagia, and dysarthria	CBS	Probable-CBS	Mother diagnosed with Pick disease	MRI: atrophy.
60, M	Expanded	Parkinsonism (hypomimia, shuffling gait, and reduced arm swing)	Possibly	Tremor, dysphagia and dysarthria, impaired memory and reduced concentration, staring expression with frontalis overactivity. Asymmetrical parkinsonism poorly responsive to L-dopa.	CBS	Probable-CBS (frontal behavioral-spatial syndrome)	No	MRI: generalized volume loss with marked involvement of the frontal and temporal lobes more severe on the left.
74, F	Intermediate allele of 27 repeats	Writing difficulties and falls	No	Parkinsonism, restriction of vertical gaze, cognitive decline, echolalia, brisk reflexes, dysphagia	Clinical PSP	Probable PSP	See pedigree (Fig. 3)	MRI: the superior cerebellar peduncles were found slender, general atrophy.

Key: CBD, corticobasal degeneration; CBS, corticobasal syndrome; F, female; M, male; MRI, magnetic resonance imaging; PD, Parkinson's disease; PSP, progressive supranuclear palsy.
