# Erratum to “The analysis of *C9orf72* repeat expansions in a large series of clinically and pathologically diagnosed cases with atypical parkinsonism” [Neurobiol. Aging 36 (2015) 1221.e1–1221.e6]

**DOI:** 10.1016/j.neurobiolaging.2015.02.027

**Published:** 2015-04

**Authors:** Lucia V. Schottlaender, James M. Polke, Helen Ling, Nicola D. MacDoanld, Arianna Tucci, Tina Nanji, Alan Pittman, Rohan de Silva, Janice L. Holton, Tamas Revesz, Mary G. Sweeney, Andy B. Singleton, Andrew J. Lees, Kailash P. Bhatia, Henry Houlden

The affiliation for Arianna Tucci in the article referenced above was listed incorrectly as, “Department of Molecular Neuroscience, UCL Institute of Neurology, The National Hospital for Neurology and Neurosurgery, London, UK”. The correct affiliation is “Division of Pathology, Fondazione IRCCS Ca’ Granda Ospedale Maggiore Policlinico, Milano, Italy” and “Department of Pathophysiology & Transplantation, Università degli Studi di Milano, Milano, Italy”.

